# The ethical dimension in published animal research in critical care: the dark side of our moon

**DOI:** 10.1186/cc13766

**Published:** 2014-03-13

**Authors:** Olivier Huet, Judy B de Haan

**Affiliations:** 1 Intensive Care Unit, Alfred Hospital, 55 Commercial Road, Melbourne 3004, VIC, Australia; 2 Baker IDI Heart and Diabetes Institute, 75 Commercial Road, Melbourne 3004, VIC, Australia

## Abstract

The replacement, refinement, and reduction (3Rs) guidelines are the cornerstone of animal welfare practice for medical research. Nowadays, no animal research can be performed without being approved by an animal ethics committee. Therefore, we should expect that any published article would respect and promote the highest standard of animal welfare. However, in the previous issue of *Critical Care*, Bara and Joffe reported an unexpected finding: animal welfare is extremely poorly reported in critical care research publications involving animal models. This may have a significant negative impact on the reliability of the results and on future funding for our research. The ability of septic shock animal models to translate into clinical studies has been a challenge. Therefore, every means to improve the quality of these models should be pursued. Animal welfare issues should be seen as an additional benefit to achieve this goal. It is therefore critical to draw conclusions from this study to improve the standard of animal welfare in critical care research. This has already been achieved in other fields of research, and we should follow their example.

## 

Since 1959, the replacement, refinement, and reduction (3Rs) guidelines have been the cornerstone of animal welfare practice for medical research. The goals of these guidelines are to protect animals during experimental research and to keep animal usage to a minimum. In the ensuing years, animal ethics has greatly progressed, and animal ethics committees assess all research projects involving animal models, and it is now impossible to perform and publish research without the approval of an animal ethics committee. Therefore, we would expect that any research involving animal models would thoroughly respect and promote animal welfare.

In the previous issue of *Critical Care*, Bara and Joffe [1] showed how ethical perspectives are reported in critical care animal research published from January to June 2012 in three major critical care journals. Seventy-seven articles were reviewed, and the authors investigated whether the description of the animal model included basic animal welfare (sample size, anesthesia, pain relief, and method of euthanasia) in the ‘Materials and methods’ section. The results are surprising yet indisputable: Only 7% of the studies reported monitoring the levels of anesthesia, 14% reported monitoring the treatment of expected pain, and less than 60% stated the method of euthanasia. Where stated, methods were found to be appropriate in only 42% of cases for the species studied. The authors also discuss the importance of correctly reporting the methods, which critically affect the reliability of the results and therefore our ability to translate fundamental research into clinical practice. Their absence may also raise concerns by funding agencies and therefore compromise the funding of critical care research.

After more than 40 years of basic research on septic shock and more than 30 phase II or III clinical trials, septic shock remains a disease without a specific treatment. There is no other such example in medicine with the incidence of this disease and its associated lethality [2]. Several hypotheses can account for this: the complexity of the immune response involved, its inter-individual variability, the different manifestations depending on the site of infection, and the timing of intervention [3]. However, before considering these complex concepts, we should make sure that the basis of our research (that is, our experimental models) is rigorous. We will not be able to overcome the challenges of treating septic shock if our most critical research tool, our animal models, is not reliable, reproducible, and relevant. These considerations are probably even more important in our area of research given the short duration over which septic shock develops (a matter of days); therefore, any event that interferes with the disease process is more likely to affect the outcome than in chronic diseases that evolve over a period of months.

Animal models of septic shock have been extensively reviewed in the past [4,5], but this is the first time that a study has addressed such methodological issues. With this study, Bara and Joffe draw attention to how important animal ethical considerations are for our research. It is a possibility that animal welfare is considered by scientists but not sufficiently reported. However, authors of scientific reports are not the only ones who should be blamed. All articles published in these high-impact factor journals are thoroughly peer-reviewed and it must therefore be assumed that reviewers have failed to point out these flaws to researchers. More importantly, this comptive analysis by Bara and Joffe indicates how poorly informed some scientists are in regards to the importance of animal welfare in their research despite the availability of guidelines [6-8]. Animal ethics applications can be very tedious, time-consuming, and laborious and, in the era of ‘publish or perish’, can appear to be futile or a waste of time. It is therefore the responsibility of ethics committees to work with scientists to increase their standards of animal welfare and thereby to increase the quality of research.

As stated by the authors, it is time for us to be more aware of the importance of the methodology used in our animal models. However, it is also time for optimism, as debate over improved animal ethics and welfare has already begun, and several reports on animal models in sepsis and welfare have already been published [9-11]. Nevertheless, these models require a greater involvement by the scientist and are time-consuming, possibly making scientists reluctant to use them. There are obviously no easy solutions, but we need to improve our experimental methods to overcome the challenge we are facing in septic shock research.

The study by Bara and Joffe has highlighted a major flaw in the way we do research; it is now our responsibility to change our experimental designs in order to improve their quality to ultimately benefit our patients. This effort has already been made in other fields of research [12,13], and we should follow their example.

### Competing interests

The authors declare that they have no competing interests.
